# Educational level, ethnicity and mortality rates in Israel: national data linkage study

**DOI:** 10.1186/s13584-021-00483-9

**Published:** 2021-08-13

**Authors:** Nehama Frimit Goldberger, Ziona Haklai

**Affiliations:** Health Information Division, Ministry of Health, Yirmiyahu, 39, 9446724 Jerusalem, Israel

**Keywords:** Mortality, Causes of death, Educational level, Educational gradient, Population group

## Abstract

**Background:**

Many studies have shown significant gaps in mortality, and cause specific mortality by educational status. This study investigated these measures in Israel by educational and ethnic status in recent decades.

**Method:**

A mortality follow-up till 2017 was done of a cohort of Israeli residents aged 25–64 in 2000 who remained in Israel and had available educational data, grouped into under 8, 9–11, 12, 13–15 and 16 and above years of education. Indirect age adjustment was used to calculate Standard Mortality Ratios (SMRs) by sex and educational group, and a Cox regression model to assess relative risk of total and cause specific mortality controlling for age and ethnic group (Jews and Others and Arabs).The analysis was repeated for each ethnic group separately.

**Results:**

2,776,422 persons were included of whom 174,792 (6.3%) died till 2017. SMR’s for total mortality of males and females with less than 8 years of education compared to 16 and over were 2.2 and 1.8, respectively. Corresponding HR were 2.13 (95% CI 2.08–2.18) and 1.77 (95% CI 1.72–1.82), respectively.

The highest cause specific hazard ratios in males were for homicide, 4.40 (95% CI 3.19–6.07), respiratory diseases, 4.01 (95% CI 3.61–4.44), infectious diseases, 3.55 (95% CI 3.15–3.19) and diabetes 3.41 (95% CI 3.06–3.79) and in females for diabetes, 4.41 (95% CI 3.76–5.16), infectious diseases, 4.16 (95% CI 3.52–4.91), respiratory diseases, 4.13 (95% CI 3.55–4.81), and heart disease, 2.96 (95% CI 2.66–3.29).

Education-adjusted risk of all-cause mortality for Arab males was 1.07 (1.05–1.09) times that of Jews and Others and non-significant in females. High mortality risk was found for Arab males and females compared to Jews and Others for homicide, diabetes, heart and cerebrovascular disease and for respiratory disease in males. Lower risk was found for suicide and infectious diseases in both sexes and

cancer in females.

**Conclusion:**

We found significant effect of educational level on all-cause and cause specific mortality, particularly respiratory diseases, infectious diseases, diabetes and homicide. Our results highlight the importance of increasing the educational level of all groups in the population and of encouraging healthy behavior in the lower educated.

**Supplementary Information:**

The online version contains supplementary material available at 10.1186/s13584-021-00483-9.

## Background

Socio-economic factors such as occupation, income and education can effect mortality, and education is a particularly useful measure, since it has a clear gradient and is measured on an interval scale.

Life expectancy by educational attainment, which reflects all-cause mortality rates, has been widely studied, included as an indicator in the Eurostat database [1], and analyzed in an OECD working paper using 2011 data [2]. In recent years, this indicator was used by the OECD together with Eurostat data to publish comparisons of 25 countries in Health at a Glance 2019, showing significant gaps in life expectancy between those with high and low education [3].

Other studies have looked at risk of mortality, CHD mortality and other cause mortality by educational level and found significant effects, such as a 32-year follow up of military inductees in the Netherlands [4], the USSR Lipid Research Clinics study which found a significantly higher risk for CHD mortality in the least educated group [5] and the Reykjavik study, which followed a large cohort in Iceland [6]. A recent study of 15 Asian cohorts found lower mortality risk with increasing education for all-cause, CVD and cancer mortality, but with differing hazard ratios (HR) [7]. A large USA study examined all cause and cause specific mortality by educational status in 2 million people of two American cancer society cohorts, and also found a clear educational gradient for HR for most causes, which had increased in many between the two cohorts [8]. Also in the USA, Hummer et al. have analyzed educational differences in mortality and discussed mechanisms involved in causing these differences [9].

Recently, changes in inequalities due to education over time have been explored in several other studies, such as that comparing years of life lost in white and black US adults between 2010 and 2017 by causes of death [10]. The English longitudinal study on aging, found mortality differences by education also when controlling for sex, age, household wealth and a health metric [11]. An international comparisons of trends in mortality inequalities by education, using data from 27 European countries was done by Mackenbach et al. [12], and by de Gelder et al. for 6 European countries, including broad groups of causes [13].

In Israel, similar studies were conducted using the Israeli longitudinal mortality studies which followed up a 20% sample of the population with educational data from the 1983 census till 1992, documented by Eisenbach et al. [14] and from the 1995 census till 2004, analyzed and compared to the former by Jaffe et al. [15]. Manor et al. reported from the former significantly lower all-cause mortality risk for Jewish males [16] and females [17] with higher education compared to the lowest group, and higher total cardiovascular (CVD) and CVD cause mortality risk in the less educated [18]. Significant changes occurred in Israel’s population between the two studies, as there were two large waves of immigration, from the Former Soviet Union (FSU) in the early 1990’s and from Ethiopia in 1984 and 1990–1991. Jaffe et al. found that although mortality rates decreased between the two studies, mortality inequalities by educational status in general increased for total, and were particularly high for CVD mortality in younger females, aged 45–64 at baseline.

These studies did not include the Israeli Arabs, who comprise about 20% of the Israeli population. Since the last study of Jaffe et al., a large decrease has occurred in cardiovascular mortality, and cancer is the leading cause of death since 2000 at most ages rather than heart disease [19].

The first aim of our study was to analyze the effect of educational level on risk of all-cause and cause specific mortality in a relatively full Israeli population cohort followed up over from 2000 until 2017. The size of the study group and long follow-up period enabled inclusion of less common causes of death, such as dementia and suicide. Would all causes show a similar educational level gradient and which would be most significantly affected by education?

The Arab population of Israel has a lower life expectancy than the Jewish population [20] and Muhsem et al. have reported on inequalities in cause specific mortality and cancer incidence [21]. A second aim of this study, which included both Jews and Others (Others are defined as non-Jews who are not Arab, about 5% of this group) and Arabs, was to check which differences would persist when controlling for education, and for which causes of death. Further, we compared educational mortality differentials by cause when each group was analyzed separately.

## Methods

### Data sources

Data on educational achievement was taken from a national database in the Israeli Central Bureau of Statistics (CBS), based on administrative reports from universities, colleges and other educational institutions, licensing registries and degree equivalence data from the Education ministry for new immigrants. This was supplemented from censuses and surveys, particularly for older people and immigrants without Israeli administrative data sources. Data from the latter sources may tend to over-estimate educational achievement due to the desire to report higher educational achievement, while administrative sources may under estimate education by missing part of the educational background. Further details on this registry are presented in the CBS publication on the effect of educational disparities on Israeli society [22].

We chose a relatively young cohort of all residents of Israel aged 25–64 in 2000, since the quality, comparativeness and completeness of the data in this registry decreases with age and we controlled for the two ethnic groups, Jews and Others and Arabs. We retained the 55–64 year old group despite missing educational data for about 20%, since they had a large number of deaths over the study period, enabling analysis of detailed causes. We did a sensitivity analysis excluding them in one regression model to see if the results changed.

Cumulative emigration data was available from 2010 onwards. We excluded those who emigrated from Israel during the years of the study and did not return until 2017, since we did not have their causes of death or exact date of emigration.

Israeli mortality data was taken from the nationwide database of causes of death prepared by the CBS, with underlying cause of death coded according to ICD-10, and were grouped into main causes (see Table 2 for codes). We linked the mortality data to the education data by the unique identification number of each Israeli resident, enabling a reliable linkage.

Education was ascertained according to years of education reported in the database divided into 5 groups: until 8 (primary school), 9–11 (partial high school), 12 (complete high school), 13–15 (higher education/first degree) and 16 and above (above first degree level). Persons with missing education data were excluded.

### Analysis

We used indirect age standardization to calculate sex specific standardized mortality ratios (SMRs). Number of persons alive each year were calculated by sex, educational group and age, grouped into 25–44, 45–54, 55–64, 65–74, and 75–84, and their cumulative total over the study period was the total age-specific person-years for each educational group. Age specific mortality rates were then calculated for each cause and for total mortality for those with 16 years education and above. These rates were used to calculate total expected deaths for each cause and for total mortality for the lower educational categories based on their total person-years by age. The SMRs were then calculated as the observed deaths divided by the expected deaths, for each sex and cause, to give the age-adjusted ratio of mortality rates in the lower educational groups compared to the highest one.

Cox regression models were used to estimate relative risks of total and cause specific mortality, adjusting for baseline age (as a continuous variable) and population group in model 1 and with the addition of education in model 2, using a separate models for each cause and sex. The time response was calculated as time in years from the beginning of the study (2000) until death, or the end of the study (2017).

In addition, we ran the age and education adjusted Cox regression model for Jews and Others and Arabs separately, to compare their relative educational risks. Education was grouped for this analysis in 3 groups (under 8, 9–12, 13 and above years) to facilitate comparison with earlier studies and since the Arab cohort was relatively small and had a small proportion in the 16 + category.

We did not have individual level data on risk factors or health behaviors. Therefore, to help explain our findings we wanted to assess the frequencies of risk factors, healthy behaviors and access to preventive measures and medical care by educational status. The CBS Social Surveys of 2010 and 2017 included a section on health and lifestyle. These surveys of about 7500 people each, aged 20 and above, are built on well-designed stratified samples interviewed face-to-face and give weighted estimates representing population characteristics. Data on a variety of health measures by educational status is readily available for download. Data we extracted from the 2017 survey (which had a larger number of measures available) and the 2010 survey [23] are presented in Additional file 1.

## Results

There were 3,018,983 residents of Israel aged 25–64 in 2000 who did not emigrate by 2017, of whom 8% had missing education data, leaving the study group of 2,776,421, 1,387,209 males and 1,389,212 females. Total person-years of follow-up was 48,640,966.

**Fig. 1:**
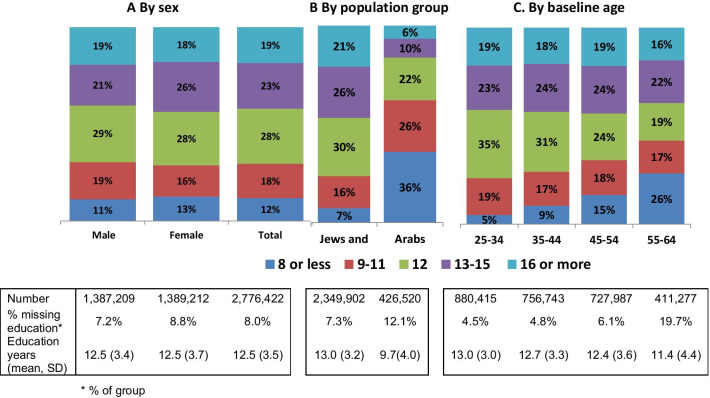
Educational status of study population by demographic characteristics. **A** By sex. **B** By ethnic group. **C** By baseline age

Figure 1 shows the educational status of the study population by demographic characteristics. Males and females had a similar educational profile, although females had more missing educational data. The largest group (28%) was those who had 12 years education followed by 23% with 13–15 years (Fig. 1A). Jews and Others had considerably more years of education than Arabs, with a mean of 13.0 years compared to 9.7, respectively. 21% of Jews and Others had 16 years of education or more compared to 6% of Arabs, and more than half of the Arabs (62%) has less than 12 years education compared to less than a quarter of the Jews and Others (23%) (Fig. 1B). The proportion of the lowest educational groups increased with baseline age (Fig. 1C) while the corresponding proportion of those with 12 years decreased. Hence, only 5% of those aged 25–34 in 2000 had primary education only, compared to 26% of those aged 55–64 in 2000, while 35% of those aged 25–34 had 12 years compared to 19% of those aged 55–64. Proportions of other educational groups differed less by age. We also see that the rate of those missing educational data increased with age, reaching a fifth (19.7%) of those aged 55–64 in 2000.

The mortality of different educational groups in shown in Table 1. There were 174,792 deaths until 2017, 108,331 of males and 66,461 of females, 6%, 8% and 5% of the study group, respectively. Mortality increased as educational years decreased, from 4% in the total 16 + group to 13% in the 8 years or less group. However, it should be noted that the lesser educated groups were also older at baseline, in particular the lowest group, mean age 48.2 compared to 41.5 in the 16 years and over group, and similar ages for the sex specific groups. Females were slightly younger at baseline in most educational groups and older at death in the lower educated.

**Table 1:** Mortality by educational status and sex, for total cohort of Israeli residents in 2000^1^ aged 25–64, followed up till end of 2017

Years of education	Number in group	Number of deaths	% mortality	Age at baseline (Mean, SD)	Age at death (Mean, SD)
*Total group*					
8 or less	328,576	42,593	13.0%	48.2 (10.4)	64.8 (9.5)
9–11	493,443	36,945	7.5%	41.3 (11.0)	60.3 (5.0)
12	789,512	39,499	5.0%	39.6 (10.3)	59.5 (11.6)
13–15	650,721	33,501	5.1%	41.8 (10.8)	62.1 (10.8)
16 or more	514,170	22,254	4.3%	41.5 (19.9)	62.7 (10.3)
Total	2,776,422	174,792	6.3%	41.8 (11.0)	61.9 (10.9)
*Males*					
8 or less	153,942	25,306	16.4%	48.4 (10.5)	64.3 (09.6)
9–11	268,448	24,944	9.3%	41.4 (11.0)	59.9 (11.4)
12	407,005	25,746	6.3%	40.0 (10.5)	59.6 (11.6)
13–15	291,383	18,150	6.2%	41.6 (10.8)	61.8 (10.9)
16 or more	266,431	14,185	5.3%	42.2 (11.1)	63.3 (10.2)
Total	1,387,209	108,331	7.8%	42.0 (11.0)	61.6 (11.0)
*Females*					
8 or less	174,634	17,287	9.9%	48.0 (10.4)	65.6 (09.4)
9–11	224,994	12,001	5.3%	41.3 (10.9)	61.2 (11.0)
12	382,507	13,753	3.6%	39.2 (10.1)	59.3 (11.5)
13–15	359,338	15,351	4.3%	41.9 (10.8)	62.4 (10.7)
16 or more	247,739	8,069	3.3%	40.6 (10.6)	61.8 (10.6)
Total	1,389,212	66,461	4.8%	41.6 (10.9)	62.3 (10.8)

^1^ Total cohort including Jews and Others and Arabs

Table 2 shows the distribution of causes of death by educational status and sex. A clear educational gradient is seen in the two leading causes of death, cancer and heart disease, with share of cancer deaths increasing with educational status, while heart disease deaths decrease. Other causes of death that also decrease with educational status are diabetes, cerebrovascular disease and respiratory disease.

**Table 2:** Causes of death by educational status, total cohort^1^ aged 25–64, 2000–2017 Percentage for each educational group

Cause of death/years of education	Total deaths for cause (N)	8 or less	9–11	12	13–15	16 or more	Total*
*Males*							
Total deaths (number)		25,306	24,944	25,746	18,150	14,185	108,331
Infectious diseases (A00-B99)	3865	4.2	3.7	3.4	3.2	2.9	3.6
Malignant neoplasms (C00-C97)	37,019	29.2	31.9	33.3	39.0	42.4	34.2
Diabetes (E10-E14)	5678	7.1	5.4	5.2	4.0	3.4	5.2
Dementia (F01-F03)	537	0.6	0.5	0.4	0.5	0.5	0.5
Heart disease (I00-I09,I11,I13,I20-I51)	15,584	15.9	14.4	13.7	13.6	13.8	14.4
Cerebrovascular disease (I60-I69)	4530	5.0	4.2	3.9	3.8	3.6	4.2
Respiratory diseases (J00-J99))	5884	8.0	5.4	5.1	4.0	3.4	5.4
Ill-defined (R95-R99)	8687	7.2	8.5	8.6	7.9	7.8	8.0
Other natural causes	17,154	17.1	15.8	15.9	15.3	14.2	15.8
Accidents (V01-X59,Y85-Y86)	4460	3.3	4.6	4.8	4.1	3.6	4.1
Suicide (X60-X84,X87.0)	2721	1.0	2.8	3.2	2.8	3.1	2.5
Homicide (X85-Y09,Y87.1)	865	0.7	1.2	1.0	0.5	0.4	0.8
Other external causes	1347	0.8	1.5	1.6	1.3	1.0	1.2
*Females*							
Total deaths (number)		17,287	12,001	13,753	15,351	8,069	66,461
Infectious diseases (A00-B99)	2304	5.1	3.9	2.9	2.5	2.3	3.5
Malignant neoplasms (C00-C97)	33,342	33.2	46.1	55.4	60.4	64.2	50.2
Diabetes (E10-E14)	3441	8.9	6.0	3.5	3.3	2.3	5.2
Dementia (F01-F03)	390	0.7	0.6	0.5	0.5	0.5	0.6
Heart disease (I00-I09,I11,I13,I20-I51)	5782	13.0	9.0	7.3	6.5	5.6	8.7
Cerebrovascular disease (I60-I69)	2531	5.4	4.0	3.1	3.0	2.8	3.8
Respiratory diseases (J00-J99))	2881	6.6	4.8	3.9	2.7	2.7	4.3
Ill-defined (R95-R99)	2775	4.2	4.7	4.2	3.9	4.0	4.2
Other natural causes	10,198	19.7	16.5	14.0	12.9	11.1	15.3
Accidents (V01-X59,Y85-Y86)	1423	2.0	2.2	2.5	2.0	2.2	2.1
Suicide (X60-X84,X87.0)	713	0.4	1.0	1.5	1.3	1.5	1.1
Homicide (X85-Y09,Y87.1)	247	0.3	0.5	0.5	0.3	0.3	0.4
Other external causes	434	0.5	0.7	0.8	0.8	0.7	0.7

^*^Not including those with missing educational status

^1^ Total cohort including Jews and Others and Arabs

The corresponding numbers of deaths and those missing education by cause are shown in Additional file 1: Table S1. Missing education was higher among the deaths, since they occurred in general in older persons.

Additional file 1: Table S2 shows percentage and numbers of causes of death stratified by population group. Similar trends were found in general for Jews and Others and Arabs, although the heart disease trend was less marked in Arabs, and they had a smaller proportion of deaths from cancer and higher proportion from diabetes than Jews and Others in all groups.

The SMRs for total mortality and causes of death are shown in Table 3 for males and females. The SMR for total mortality showed rates 2.2 times higher for males with 8 years education or less compared to 16 + and 1.8 times for females, decreasing to 1.3 and 1.2, respectively, for 13–15 years compared to 16 +.

**Table 3:** Age-Standardized Mortality Ratios (SMRs) by cause of death, with 95% confidence interval (CI), aged 25–64 year old at baseline^1^, 2000–2017

Cause of death/Years of education	8 or less		9–11		12		13–15	
	Compared to 16 and over							
	SMR	95% CI	SMR	95% CI	SMR	95% CI	SMR	95% CI
*Males, N = 1,387,209*								
Infectious diseases	2.9	(2.7–3.1)	2.6	(2.4–2.7)	1.8	(1.7–2.0)	1.4	(1.3–1.6)
Malignant neoplasms	1.5	(1.4–1.5)	1.5	(1.4–1.5)	1.2	(1.2–1.2)	1.2	(1.2–1.2)
Diabetes	4.3	(4.1–4.5)	3.2	(3.0–3.4)	2.5	(2.3–2.6)	1.6	(1.5–1.7)
Dementia	1.9	(1.6–2.3)	2.0	(1.7–2.4)	1.5	(1.2–1.8)	1.4	(1.1–1.7)
Heart disease	2.4	(2.4–2.5)	2.1	(2.0–2.1)	1.5	(1.5–1.6)	1.3	(1.2–1.3)
Cerebrovascular disease	2.8	(2.6–2.9)	2.3	(2.2–2.5)	1.7	(1.6–1.8)	1.4	(1.3–1.5)
Respiratory diseases	4.4	(4.2–4.6)	3.2	(3.0–3.4)	2.5	(2.3–2.6)	1.5	(1.4–1.7)
Ill-defined	2.2	(2.1–2.3)	2.1	(2.0–2.2)	1.5	(1.5–1.6)	1.2	(1.2–1.3)
Other natural causes	2.5	(2.4–2.6)	2.2	(2.1–2.3)	1.7	(1.7–1.8)	1.4	(1.3–1.4)
Accidents	2.6	(2.4–2.7)	2.4	(2.2–2.5)	1.7	(1.6–1.8)	1.4	(1.3–1.5)
Suicide	*0.9*	*(0.8–1.0)*	1.7	(1.5–1.8)	1.3	(1.2–1.4)	1.1	(1.0–1.2)
Homicide	5.9	(5.0–6.9)	5.7	(5.1–6.4)	3.1	(2.7–3.5)	1.4	(1.1–1.7)
Other external causes	2.3	(2.0–2.7)	2.7	(2.4–3.0)	2.0	(1.8–2.2)	1.5	(1.3–1.7)
All cause mortality	2.2	(2.1–2.2)	2.0	(1.9–2.0)	1.5	(1.5–1.5)	1.3	(1.3–1.3)
*Females, N = 1,389,212*								
Infectious diseases	3.4	(3.2–3.7)	2.6	(2.4–2.8)	1.7	(1.5–1.9)	1.2	(1.1–1.3)
Malignant neoplasms	*1.0*	*(1.0–1.0)*	1.1	(1.1–1.2)	1.1	(1.1–1.1)	1.1	(1.1–1.1)
Diabetes	5.8	(5.5–6.1)	3.9	(3.7–4.2)	2.1	(1.9–2.3)	1.6	(1.5–1.8)
Dementia	1.7	(1.4–2.1)	1.7	(1.3–2.1)	1.5	(1.2–1.9)	*1.1*	*(0.9–1.4)*
Heart disease	3.5	(3.3–3.6)	2.4	(2.3–2.6)	1.8	(1.7–1.9)	1.3	(1.2–1.4)
Cerebrovascular disease	3.0	(2.8–3.2)	2.2	(2.0–2.4)	1.5	(1.4–1.7)	1.2	(1.1–1.3)
Respiratory diseases	3.7	(3.5–3.9)	2.7	(2.5–3.0)	2.0	(1.8–2.2)	1.1	(1.0–1.2)
Ill-defined	1.9	(1.8–2.1)	1.8	(1.7–2.0)	1.3	(1.2–1.5)	1.1	(1.1–1.2)
Other natural causes	2.9	(2.8–3.0)	2.3	(2.2–2.4)	1.7	(1.6–1.7)	1.3	(1.3–1.4)
Accidents	2.0	(1.8–2.3)	1.6	(1.4–1.8)	1.4	(1.2–1.5)	1.2	(1.0–1.3)
Suicide	0.8	(0.6–0.9)	*1.1*	*(0.9–1.3)*	1.2	(1.0–1.3)	*1.1*	(0.9–1.2)
Homicide	2.7	(2.0–3.5)	2.6	(2.0–3.4)	1.7	(1.3–2.1)	*1.1*	*(0.8–1.5)*
Other external causes	1.9	(1.5–2.4)	1.6	(1.3–2.0)	1.3	(1.1–1.6)	1.5	(1.2–1.8)
All cause mortality	1.8	(1.8–1.8)	1.6	(1.5–1.6)	1.3	(1.3–1.3)	1.2	(1.2–1.2)

^1^Total cohort including Jews and Others and Arabs. Italicized numbers: SMR not significant

The SMR for males with up to 8 years of education compared to 16 + was highest for homicide (5.9) followed by respiratory diseases (4.4) and diabetes (4.3). In females, diabetes was highest (5.8) and then respiratory diseases (3.7) and heart disease (3.5). Infectious diseases and cerebrovascular disease followed in the ranking for both sexes. Lowest was suicide, insignificant for males, and showing a lower mortality for females (0.8) among those with 8 years or less. Cancer, too, had low SMRs, although higher in males than females. Similar ranking was found for other educational groups compared to 16 +, and in most the SMR decreased with education.

### Arabs compared to Jews and others

Table 4 shows the results of the Cox regression models. In model 1 controlling for age only, Arabs had a higher risk of mortality than Jews and Others for most causes of death, with the exception of suicide in both sexes and cancer in females.

**Table 4:** Hazard ratios from cox regression model predicting risk of death by cause with 95% confidence interval (CI), 25–64 year old at baseline

	Model 1			Model 2												
Cause of death/Effect	Population group			Population group		Education										
	Arabs			Arabs		8 or less		9–11			12			13–15		
	v Jews and Others			v Jews and Others		v 16 and over										
	HR	95% CI	HR		95% CI	HR	95% CI		HR	95% CI		HR	95% CI		HR	95% CI
*Males, N = 1,387,209*																
Infectious diseases	*0.95*	*0.86–1.05*	0.65		0.58–0.72	3.55	3.15–3.99		2.60	2.31–2.92		1.77	1.57–1.99		1.42	1.25–1.61
Malignant neoplasms	1.09	1.06–1.12	*0.97*		*0.94–1.01*	1.45	1.40–1.51		1.47	1.42–1.52		1.19	1.15–1.23		1.17	1.14–1.22
Diabetes	2.36	2.22–2.50	1.72		1.61–1.84	3.41	3.06–3.79		2.96	2.66–3.29		2.37	2.14–2.64		1.54	1.37–1.73
Dementia	*0.80*	*0.60–1.08*	0.63		0.46–0.85	2.21	1.65–2.96		2.06	1.54–2.76		1.44	1.07–1.94		1.38	1.01–1.87
Heart disease	1.79	1.72–1.86	1.44		1.38–1.51	2.07	1.95–2.19		1.95	1.85–2.06		1.49	1.41–1.57		1.25	1.18–1.33
Cerebrovascular disease	1.56	1.45–1.69	1.19		1.10–1.29	2.55	2.29–2.84		2.27	2.04–2.52		1.69	1.52–1.88		1.36	1.22–1.53
Respiratory diseases	2.14	2.01–2.27	1.44		1.35–1.54	4.01	3.61–4.44		3.01	2.72–3.35		2.31	2.09–2.57		1.49	1.33–1.68
Accidents	1.37	1.27–1.47	*1.06*		*0.98–1.15*	2.65	2.36–2.98		2.31	2.08–2.57		1.65	1.49–1.83		1.36	1.21–1.52
Suicide	0.34	0.29–0.40	0.30		0.26–0.35	1.53	1.30–1.79		1.85	1.64–2.08		1.28	1.14–1.44		*1.08*	*0.95–1.23*
Homicide	3.26	2.84–3.74	2.31		1.99–2.68	4.40	3.19–6.07		4.53	3.38–6.07		2.74	2.05–3.68		*1.38*	*0.98–1.95*
All cause mortality	1.33	1.31–1.36	1.07		1.05–1.09	2.13	2.08–2.18		1.91	1.87–1.95		1.47	1.44–1.50		1.26	1.23–1.29
*Females, N = 1,389,212*																
Infectious diseases	1.44	1.28–1.62	0.79		0.69–0.90	4.16	3.52–4.91		2.68	2.26–3.18		1.65	1.38–1.96		1.25	1.05–1.50
Malignant neoplasms	0.81	0.78–0.84	0.83		0.80–0.86	*1.03*	*0.99–1.07*		1.14	1.10–1.18		1.09	1.06–1.13		1.12	1.08–1.16
Diabetes	3.24	3.00–3.50	1.93		1.76–2.10	4.41	3.76–5.16		3.71	3.16–4.36		1.99	1.68–2.35		1.59	1.35–1.88
Dementia	*0.80*	*0.60–1.08*	*0.73*		*0.49–1.07*	1.87	1.30–2.69		1.67	1.13–2.45		1.48	1.01–2.17		*1.10*	*0.75–1.60*
Heart disease	2.41	2.26–2.57	1.58		1.47–1.70	2.96	2.66–3.29		2.34	2.10–2.62		1.71	1.53–1.91		1.30	1.16–1.45
Cerebrovascular disease	2.10	1.90–2.32	1.39		1.24–1.56	2.69	2.30–3.14		2.17	1.85–2.54		1.50	1.28–1.76		1.21	1.03–1.43
Respiratory diseases	1.63	1.47–1.80	*0.91*		*0.81–1.02*	4.13	3.55–4.81		2.77	2.36–3.24		1.94	1.66–2.28		*1.16*	*0.98–1.37*
Accidents	1.29	1.12–1.50	*0.96*		*0.82–1.13*	2.11	1.73–2.57		1.63	1.35–1.98		1.36	1.13–1.63		*1.16*	*0.96–1.40*
Suicide	0.32	0.23–0.45	0.30		0.21–0.42	*1.31*	*0.97–1.78*		*1.26*	*0.98–1.62*		*1.18*	*0.94–1.48*		*1.12*	*0.89–1.40*
Homicide	2.10	1.58–2.79	1.51		1.09–2.08	2.52	1.49–4.25		2.43	1.52–3.87		*1.57*	*1.00–2.47*		*1.16*	*0.71–1.89*
All cause mortality	1.27	1.25–1.30	*1.01*		*0.99–1.04*	1.77	1.72–1.82		1.55	1.50–1.59		1.28	1.24–1.31		1.17	1.14–1.20

Model 1: Adjusting for age and population group only, Model 2: Adjusting for age, population group and education

All HR significant with *p* ≤ 0.05 except italicized numbers

Relative risk of all-cause mortality for Arab males compared to Jews and Others was reduced by adding education to the model (model 2) from 1.33 (95% CI 1.31–1.36) to 1.07 (95% CI 1.05–1.09) and from 1.27 (95% CI 1.25–1.30) to 1.01 (95% CI 0.99–1.04) in females, non-significant. Other HR’s for Arabs compared to Jews and Others were also reduced by adding educational level to the model, with particularly large decreases, over 25%, in relative risk for respiratory disease, homicide and diabetes in both sexes, and also for infectious, cerebrovascular and heart diseases in females. The lowest effect of adding education was found for suicide and cancer, where the HR actually increased slightly for females.

Particularly high mortality risks for Arabs remained in model 2 for males from homicide 2.31 (95% CI 1.99–2.68) and diabetes 1.72 (95% CI 1.61–1.84), followed by respiratory and heart diseases (1.44), while the risk was low for suicide (0.30), dementia (0.63) and infectious diseases (0.65). Relative risk for cancer and accidents was not significant in model 2. In Arab females, the highest relative risk was from diabetes, 1.93 (95% CI 1.76–2.10), heart disease 1.58 (95% CI 1.47–1.70), followed by homicide (1.51) and cerebrovascular disease (1.39), while a low risk compared to Jews and Others was found for suicide (0.30), infectious diseases (0.79) and cancer (0.83).

### Educational effects

We found steadily decreasing HRs for increasing educational categories compared to the highest for almost all causes. The risk of all-cause mortality for males in the lowest educational category (up to 8 years) was 2.13 (95% CI 2.08–2.18) times that of the highest (16 + years), and 1.77 (95% CI 1.72–1.82) times for females. The HR for the following educational groups, 9–11, 12 and 13–15 years compared to 16 + years steadily decreased from 1.91 (95% CI 1.87–1.95) to 1.26 (95% CI 1.23–1.29) for males, and 1.55 (95% CI 1.50–1.59 to 1.17 (95% CI 1.14–1.20) for females, respectively.

The highest cause specific hazard ratio (HR) for the highest compared to lowest education was found in males for homicide 4.40 (95% CI 3.19–6.07) and in females for diabetes 4.41 (3.76–5.16). Respiratory diseases were next highest in males 4.01 (95% CI 3.61–4.44) followed by infectious diseases and diabetes, while also the female HRs were high for infectious and respiratory diseases, followed by heart disease.

Cancer had the lowest HRs of causes, 1.45 (95% CI 1.40–1.51), and 1.47 (95% CI 1.42–1.52) for the two lowest educational levels in males and non-significant for the lowest level in females, and 1.1 for other levels. Suicide, too, did not show the educational gradient of other causes and the HR was insignificant in females.

We tested the proportional hazard assumption of the Cox regression by adding linear time interactions with the educational groups and population group to the models. Some of the time interactions were significant showing violation of the PH assumption in these models, for example for all-cause mortality in males for all education groups and population group, and in females for the 2 higher education groups and for population group. The parameter estimates of these time interactions with educational groups was negative, implying reduction in HR over time while that for the population group interactions was positive implying an increase in some HR’s over time, as explained by Bellera et al. [24]. We used these time-dependent parameters to calculate the HR’s at various times along the study period. Although the HR’s changed, the significant educational trends at each point in time remained. We conclude that although the HRs presented in Table 4 may be an approximate average over the time period, the trends we found are correct.

### Educational effects in separate models for Jews and Others and Arabs

**Fig. 2:**
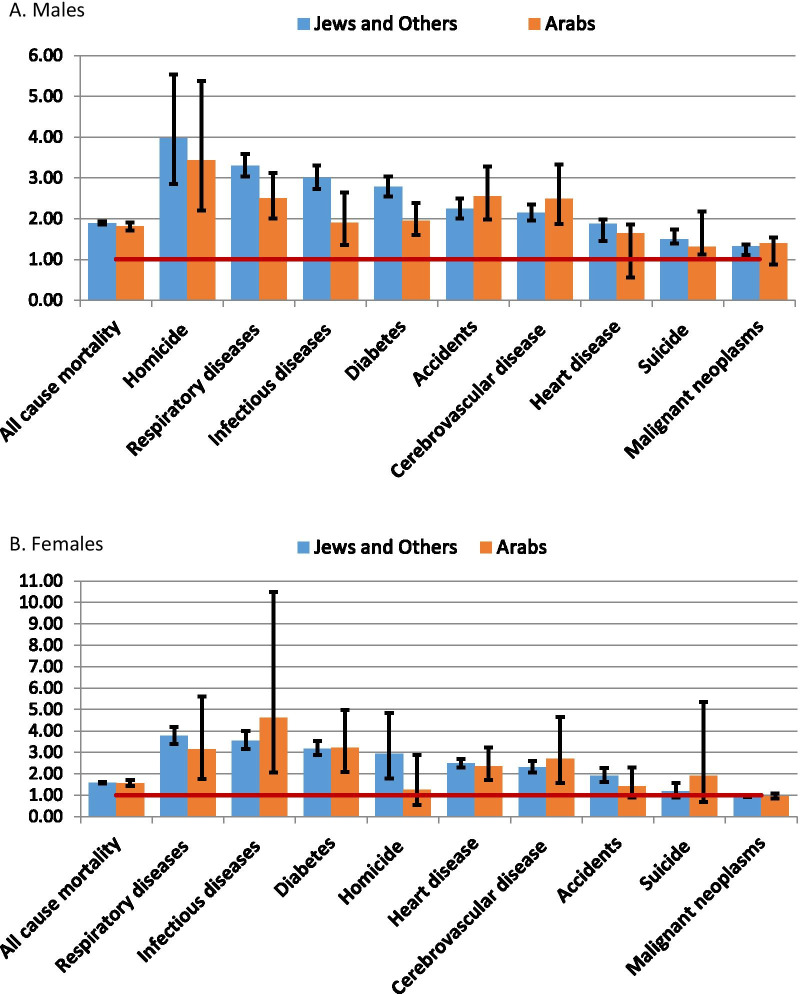
Comparison of hazard ratios for Jews and Others and Arabs by cause of death, 13 years and over compared to 8 or less (with 95% CI). **A** Males. **B** Females

A comparison of HRs for up to 8 years of education compared to 13 years and above for the separate age adjusted models of Jews and Others and Arabs is shown in Fig. 2 and detailed results for the 2 educational group HRs with CI are in Additional file 1: Table S3. We note almost identical HRs for all-cause mortality in both sexes and for cancer and diabetes mortality in females, and similar ones and similar ranking for most causes. An exception is the HRs for diabetes and infectious diseases in males which were higher for Jews and Others than Arabs.

Additional file 1: Table S4 shows the results of the regression models in `a younger cohort aged 25-54 at baseline, who had little missing educational data. All-cause mortality and cause specific mortality showed a similar educational gradient, but with higher HRs, in particular for the lowest educational categories. Female Arabs had a lower risk of all-cause mortality (HR = 0.88, 0.85-0.91) while males were not significantly different from Jews and Others.

Additional file 1: Table S5 shows data from the Social Surveys of 2017 and 2010. We see a clear relationship with education for many risk factors and health behaviors. For example the lesser educated are more obese, smoke and are exposed to smoke more, exercise and are dieting to reduce weight less, drink more sweet drinks and eat less vegetables. However, eating whole-grain food was surprisingly more frequent in lower educational categories.

The lesser educated also had lower access to health care, such as less supplementary health insurance and more prescription medications not bought. Preventive medicine such as mammograms and occult blood tests did not differ greatly by educational status, but PAP smears were much more frequent in the highly educated.

## Discussion

Our study found increased mortality as educational years decreased, particularly high differentials for respiratory diseases, infectious diseases, diabetes and homicide and lower for cancer and suicide. Arabs had similar educational differences as Jews and Others, and lower risk of mortality for suicide, cancer in females, infectious diseases and dementia in males, and significantly higher risk for diabetes, homicide, heart and cerebrovascular diseases, and respiratory diseases in males, after controlling for educational status.

How does education affect morbidity and mortality? Phelan et al. [25] suggest multiple mechanisms for the effect of socio-economic status on health, such as influencing disease outcomes, through multiple risk factors and affecting access to resources that can be used to minimize risk factors or treat existing disease. Let us look at whether our findings confirm education as performing these mechanisms showing it to be a correct social determinant of health in Israel.

Firstly, we saw (Additional file 1: Table S5) that the more educated in general had a healthier lifestyle and lower risk factors for disease, probably due to better knowledge and the economic ability to act accordingly. They smoked less, had lower BMI, were on weight reducing diets more often, ate more vegetables, drank less sweet drinks and did more physical exercise (although there were some exceptions to these trend for the lowest educational group). These differences in behavior appear to be reflected in the high HRs we found for the more ‘preventable’ diseases of diabetes, heart disease and cerebrovascular disease, and similar to the high proportion of premature deaths for these diseases attributable to socio-economic inequalities found by Lewer et al. [26], and strongest educational gradient found by Steenland et al. [8].

Educational level has economic consequences, with higher education giving better employment opportunities, income, housing, and access to healthcare, shown by the increasing rates of supplementary health insurance with education. Although Israel has universal health insurance that covers most health care, we see that the lower educated reported more often doing without prescription medicine or having medical treatments, which could contribute to worse outcomes for many diseases. Crowded housing may lead also to more infectious diseases, for which we found a high HR.

As noted by Hummer et al. [9], we saw a strong influence on causes linked with social and behavioural risk factors, such as homicide and respiratory diseases, the causes with the highest HR in males.

A lesser effect of education was found on causes less amenable to control and prevention, such as cancer. In addition, some screening tests for cancer, such as mammograms and testing for faecal occult blood are covered by the Israeli health ‘basket’ of services, and recent health policy encourages their implementation. Indeed, Additional file 1: Table S5 showed little difference in frequency of mammograms between the educational groups, and the occult blood test was more frequent at lowest educational level, although this could be because the better educated used colonoscopies instead, more frequent among them. The PAP smear test, however, does show the educational gradient, but may be less important for the lower educational groups, in view of their lifestyle. Breast cancer, in particular, has been reported higher in the higher educated, although Trewin et al. have shown the mortality trend to have begun reversing in Norway [27]. Nevertheless, as the leading cancer in women, this may contribute to the lack of significant difference found for cancer mortality among lowest educated women. The relatively low HR for cancer is similar to that found in other studies, such as Yang et al. [7].

There may also be an effect of greater work stress on those with higher education which may offset some of their advantage, as reported in a meta-analysis by Yang et al. that some cancers, such as colorectal and lung, were associated with work stress [28]. We also note, for example, that the higher educated ate 2 or more portions of wholegrain food less frequently than the lesser educated.

Suicide is one cause that seems to be lesser affected by educational status, and in females, the effect is non-significant.

We thus see that our study supports education as a social determinant of health. The value of education as a socio-economic indicator is also since it is usually attained early in life and more likely to be a cause of better health than income or occupation which may have a two-way relationship with heath status [3]. In Israel, a disadvantage of education as a measure is that some of the population may have had limited opportunity for formal education due to the effect of the Holocaust, World War II or immigration from other countries, but nevertheless achieved high socio-economic status through informal education, or despite this lack of education. In addition, it is difficult to quantify the effect of religious studies, such as in Yeshivot, which do not enter the regular educational attainment measures. Social capital can also offset low formal educational achievements, which can lead to better health outcomes, as reported by Chernikovsy et al. [29].

### Arabs compared to Jews and Others

Life expectancy at all ages has been higher for Jews than Arabs throughout the years and our age-adjusted model found significantly higher all-cause mortality and for mortality from most causes for Arabs. However, we found that after controlling for educational status too, there was only a slightly higher all-cause mortality risk for Arab males (HR = 1.07, 95% CI 1.05–1.09), and a non-significant comparative risk for females while for the younger cohort, aged 25–54 in 2000 (Additional file 1: Table S3), the adjusted mortality risk was lower for Arab females than Jews and Others (HR = 0.88, 95% CI 0.85–0.91) and insignificant for males. This would suggest that much of the difference in overall mortality between Jews and Others and Arabs can be attributed to educational differences. The effect of education was particularly strong in mitigating differences for respiratory diseases, homicide and diabetes and even more causes in females.

When we looked at the cause specific risks of Arabs compared to Jews and Others, we see differences even after adjusting for education, such as with much higher mortality from diabetes, particularly in females, heart disease, and homicide and respiratory disease in males—the latter reflecting the high smoking rate among Arab males [30]. These high risk causes are partially offset by significantly lower risks for suicide, dementia in males, infectious diseases, and cancer in females. The lower risk for cancer in Arab females reflects their lower risk for breast cancer [30], the leading female cancer. However, since Arab males have a higher incidence of lung cancer [30], the leading cancer for male mortality, probably also due to their high smoking levels, we found their comparative risk for cancer mortality compared to Jews and Others was insignificant.

Therefore, we would suggest that increasing educational level can be an effective intervention to improve Arab health, but this needs to be supplemented by ethnically appropriate programs targeting high smoking in males and high diabetes rates.

### Comparison with previous Israeli studies—changes over time

Jaffe et al. [15] reported an increase in educational disparities between the lowest and highest educated groups, between census cohorts of Jews and Others aged 25–64 from 1983 and 1995, for all-cause and particularly for CVD mortality in females. In a similar group of Jews and Others (Fig. 2, Additional file 1: Table S3), we did not find a further increase in our 17 year follow-up from 2000, but rather our HRs were lower than the ORs of Jaffe et al., 1.89 (95%CI 1.86–1.93) for all-cause mortality for males and 1.58 (95%CI 1.86–1.93) for females, compared to 2.09 (95%CI 1.91–2.28) 2.02 (95%CI 1.81–2.25), respectively, found by Jaffe et al. Although our HRs for CVD were higher for females than males, 2.49 (95%CI 1.30–2.69) for heart disease and 2.30 (95%CI 1.30–2.69) for cerebrovascular disease they were less than half what Jaffe et al. found for CVD mortality, 5.14 (95%CI 3.48–7.60).

The narrowing of educational disparity particularly for CVD compared to Jaffe et al. is good news. We would suggest that the reason may be due to the steep decline in CVD as leading causes of death [19] over the period of our study, which was just beginning at the end of Jaffe et al.’s follow-up of causes of death in 2000. This was fueled by increasing awareness and treatment of risk factors, such as hypertension and hyperlipidemia, and better interventions and treatment for CVD, which became somewhat more accessible and available to those of lower socio-economic and educational levels than in 1995–2000. We hope this trend will continue to further reduce the gap in CVD mortality between the higher educated and the lower.

### Strengths and limitations

The strength of this study is the large nationwide cohort followed up for 17 years, allowing analysis of educational differences in mortality for a range of causes of death with significant results.

The first limitation of our study was the missing data for education, particularly at older ages. Missing educational data has been reported as a problem in other studies too [3], and can be addressed in two ways. The persons with missing data are sometimes assigned to the lowest educational category, which could be supported in our study by their cause of death profile (Additional file 1: Table S1), similar to that of the lowest educated group. This method was adopted by the CBS in their publication [22], which includes results for mortality in our cohort, similar to those we found. In this paper, we chose the other, perhaps more exact way of handling them, by excluding them from the study, which might lead to an under-estimation of the lower educated. However, when we checked a younger cohort which had a smaller number missing education, as a sensitivity analysis, we found similar results.

A second limitation was not having individual level data on risk factors and economic and health indicators, such as smoking, hypertension, hyperlipidemia, income and occupation, which could have been mediators for the educational effects we found, as found by Steenland et al. [8].

Although we did multiple comparisons, due to our large study cohort, most of our results were highly significant (*p*-value < 0.0001), and would remain significant after correction for multiple comparisons.

Another limitation is that the proportional hazard assumption was not satisfied for all covariates in all models. However, the changes over time indicated by the estimated parameters for the time dependent variables that were significant did not lead to any major changes in our findings.

### Health policy implications and recommendations

We found higher mortality in those with lower educational status. This needs to be addressed in two ways. Firstly, increasing the educational level of the population should help improve population health. Secondly, health-promoting interventions need to be provided in particular to the lesser educated, encouraging smoking cessation, eating a healthy diet and exercising regularly. The educational level in Israel has been increasing: according to the Statistical Abstract of the CBS [31] those with 13 years or more of education increased from 45 to 55% for Jews and Others between 2005 and 2017 and from 19 to 26% for Arabs. It is to be hoped that improved education will help close the health gap in Israel, but needs to be encouraged in all population groups to reduce future inequalities.

## Conclusion

We found large differences in mortality between those with different educational levels, particularly high for diabetes, homicide, respiratory and infectious diseases, but lower for cancer and suicide. Education has been recently called in a Lancet editorial ‘the most important modifiable social determinant of health’ [32]. Our results highlight the importance of increasing the educational level of all groups in the population and of encouraging healthy behavior in the lower educated.

## Supplementary Information

Additional file 1. Supplementary tables for paper_new_3. **Table S1**: Causes of death by educational status and sex (numbers of deaths)**Table S2**: Causes of death by educational status and population group (percentage and numbers of deaths). **Table S3**: Hazard ratios from separate cox regression models predicting risk of death by cause for Jews and Others and Arabs aged 25-64 at baseline with 95% confidence interval (CI). **Table S4**: Hazard ratios from cox regression model predicting risk of death by cause with 95% confidence interval (CI) aged 25-54 at baseline.**Table S5**: Health influencing factors from the Social Surveys of 2017 and 2010 by educational status (percentage)

## Data Availability

The data file used for this analysis was prepared by the CBS from national databases and is only available for use in their research room, due to privacy considerations.

## Abbreviations

BMI: Body Mass Index
CBS: Central Bureau of Statistics
CI: Confidence Interval
CVD: Cardiovascular disease
HR: Hazard Ratio
ICD-10: International Classification of Diseases-Tenth Edition
SMR: Standard Mortality Ratios
